# Potential molluscicidal and antimicrobial activities of rare earth elements against the land snail *Theba pisana* and certain microorganisms

**DOI:** 10.1038/s41598-025-07169-1

**Published:** 2025-06-25

**Authors:** Hesham A. M. Ibrahim, M. G. Fadl, Ibrahim A. I. Hassan, Mohammed E. Gad, Soma A. El Mogy, Mona M. Khalifa, A. I. L. Abd El Fatah

**Affiliations:** 1 Department of Agricultural Zoology and Nematology, Faculty of Agriculture, Al-Azhar University, Assiut Branch, Assiut, 71524 Egypt; 2 Nuclear Materials Authority, Production sector, Reactors Materials Department, El Maadi, P.O. Box 530, Cairo, Egypt; 3 Department of Chemistry, South Valley University, Qena, 83523 Egypt; 4 Department of Zoology and Entomology, Faculty of Science, Al-Azhar University, Nasr City, Cairo, 11884 Egypt; 5 Materials Testing and Surface Chemical Analysis Lab, National Institute of Standards (NIS), Giza, Egypt; 6 Department of Biochemistry, College of sciences, Cairo University, Giza, Egypt

**Keywords:** Rare Earth, Land snail, Antimicrobial, Microorganism, *Theba pisana*, Biological techniques, Microbiology, Chemistry, Materials science

## Abstract

A novel application of light rare earth elements (LREEs) was explored for their biological activity as potential eco-friendly molluscicides and antimicrobials on *Theba pisana* (Müller, 1774) and their feeding behavior, and microorganisms like *Candida albicans*, *Bacillus cereus*, *Aspergillus niger*, *Staphylococcus aureus*, and *Escherichia coli*. Our data showed increased snail mortality with higher element concentrations till 500 mg/L. LC_25_ and LC_50_ values after ten days of exposure were 513.70 and 3012.72 mg/L, respectively, lower survival rates than the control. As treatment concentration and exposure duration increased, the ingested leaf area and daily consumption rates of treated lettuce leaves declined. On day one, consumption dropped from 60.00 ± 0.00 cm² (control) to 30.25 ± 6.13 cm² at 500 mg/L, further decreasing to 31.25 ± 0.76 cm² and 14.00 ± 1.46 cm² by day four at the same concentrations. Low concentrations had minimal impact on snail feeding, while higher levels significantly reduced appetite, consumption and freshness of leaves. LREEs-based formulations exhibited marked antimicrobial activity against all tested pathogens by the measured inhibition zones. Results highlight the promising application of LREEs in integrated pest and microbial disease management. However, these findings warrant further investigation to optimize their safe and practical use in the field.

## Introduction

Land snails have rapidly increased over the last few years and several species have been identified in numerous Egyptian governorates^1–3^. Snails are classified as mollusks (Class: Gastropoda), and they can be found in marine^4^ freshwater^5,6^ and terrestrial habitats^7^. *Theba pisana* (Müller, 1774) (Gastropoda: Helicidae), a white garden snail from the Helicidae family of gastropods, is recognized as an invasive terrestrial species that has proliferated across various Mediterranean nations. The small size, longevity, and rapid reproduction rates of *T. pisana*, which can yield up to 3,000 snails on a tree in under five years, contribute to its significant threat^8^. The helicid white garden snail, *T. pisana*, is one of several species of herbivorous land snails that are used as a sensitive indicator for diagnosing chemical contamination and climate change. The potential application of *T. pisana* as a model organism in laboratory toxicity assays and biomonitoring studies, serving as a bioindicator for metal and organic soil contamination, is fully documented^9,10^. Radwan and Gad^11^ indicated that the land snail *T. pisana* exhibits clear biochemical responses when exposed to Abamectin, affecting energy reserves and enzyme activities. These findings suggest that this species can serve as a sensitive bioindicator for monitoring environmental pollutant toxicity. Land snails are considered to be major pests since they cause considerable damage to many parts of plants and attack a wide range of crops^12^. These gastropods attack plants at different stages of growth, which reduces the amount of produce they can yield. The primary defense against land snails is chemical molluscicides; however, due to their toxicity to both aquatic and terrestrial organisms, there is increasing interest in identifying suitable ecologically eco-friendly molluscicides^13–16^.

The lanthanide series, which is made up of 15 elements on the periodic table, along with scandium (Sc) and yttrium (Y), are known as rare earth (RE) elements. They are categorized into two groups according to their ionic radius: light rare earth elements (LREEs) ranging from La to Sm and heavy rare earth elements (HREEs) spanning from Eu to Lu. From a specific viewpoint, these metals have comparable chemical and physical properties and universally originate from the same mineral assemblages. Over 200 minerals encompass rare earth elements (REEs), such as monazite, xenotime, bastnäsite, apatite, and zircon. REE-rich mineral species are extensively dispersed throughout various geological settings. The primary forms of REE deposits are REEs linked with carbonatites and alkaline igneous rocks, heavy mineral sands (e.g. monazite-xenotime), and supergene ion-adsorption clay deposits. Monazite and bastnäsite are two of the most cost-effective minerals that are rich in rare earth elements (REE). Monazite is one of the most abundant beach sand minerals found in vein deposits and acidic igneous rocks which containing mainly 20 − 30% Ce_2_O_3_, 4 − 12% ThO_2_ and 10 − 40% La_2_O_3_^17^. Due to their specific spectroscopic and magnetic characteristics, rare earth elements (REEs) are essential in various fields, including industrial applications and advanced material sciences, such as electric and hybrid vehicles, electrical components, metal alloys, vital military uses, fluorescent lighting, and the production of high-intensity magnets^17^. Because these elements are readily available from the environment, it is conceivable that organisms may encounter and interact with the RE elements during their life. Indeed, plants and fungi grown in various regions of the world accumulate several kinds of lanthanides, and bacteria, cultured in the presence of REE, also accumulate these elements. RE elements are known to have antibacterial activities and many physiological effects on mammalian cells when they complex with organic compounds. However, systematic studies on the antibacterial activities of inorganic RE salts are scarce to date. Various researchers have investigated the direct digestion of monazite sand produced from black sands utilizing acidic solutions of sulfuric, hydrochloric, and nitric acids under a range of experimental conditions. Sulfuric acid is predominantly utilized for the digestion of monazite, where the sulfate (SO4^2−^) ion of H_2_SO_4_ functions as a ligand that interacts with rare earth elements at elevated temperatures.

The separation of rare earth elements is a challenging process in metallurgical extraction. The separation procedure for rare earth elements (REEs) entails multiple treatments due to their chemical similarities, including the hydrometallurgical method. The extraction of rare earth elements (REEs) from ores is a multifaceted and labor-intensive procedure that encompasses beneficiation, mineral concentrate breakdown for REE extraction, and chemical processing involving impurity elimination and individual REE separation to yield a pure, marketable product. The hydrometallurgical method encompasses multiple procedures, including (i) roasting or calcining the REE concentrate; (ii) leaching, neutralization, and precipitation techniques; and (iii) separation and purification operations using solvent extraction and ion exchange^18–24^. Rare earth elements (REEs) are critical for our modern lifestyles and the transition to a low- carbon economy. Recent advances in our understanding of the role of REEs in biology, particularly methylotrophy, have provided opportunities to explore biotechnological innovations to improve REE mining and recycling. In addition to bacterial accumulation and concentration of REEs, biological REE binders, including proteins (lanmodulin, lanpepsy) and small molecules (metallophores and cofactors) have been identified that enable REE concentration and separation. REE-binding proteins have also been used in several mechanistically distinct REE biosensors, which have poten- tial application in mining and medicine. Notably, the role of REEs in biology has only been known for a decade, suggesting their considerable scope for developing new understanding and novel applications^25^. Metal and metal oxide nanoparticles, including Ag, Au, ZnO, TiO_2_, CuO, and Fe_2_O_3_, are currently receiving more and more attention for their antibacterial capabilities. This study explores the innovative application of light rare earth elements (LREEs) as potential molluscicides and antimicrobial agents in controlling *T. pisana* and inhibiting microbial pathogens, *Candida albicans*, *Bacillus cereus*, *Aspergillus niger*, *Staphylococcus aureus*, and *Escherichia coli*, addressing a critical gap in sustainable pest and disease management strategies.

## Methods

### Experimental snails gathering

Individual’s adult snails of *Theba pisana* were obtained from different infested orchards at Faisal district, Suez governorate, Egypt (30°06’22.0"N 32°31’34.3"E). The collected snails were moved to the laboratory of the Agricultural Zoology and Nematology Department at the Faculty of Agriculture, Al-Azhar University, Assiut Branch. These snails were reared under laboratory conditions at a temperature of 25 C◦±2 C◦ and relative humidity of 75% ±5% in plastic containers and nurtured by fresh lettuce leaves for 14 days before treatment for acclimatization, and the boxes were wrapped in muslin and secured with a rubber band to prevent snails from escaping. Dead and unhealthy snails were removed, and only healthy ones were used in the experiments^26^.

### Tested materials

The unique hydrothermal autoclave process involves dissolving monazite ore produced from Egyptian beach black sands with sulfuric acid^27^. The procedure was conducted on a 100 g sample of monazite using concentrated H_2_SO_4_. Approximately 85% of the sample was solubilized, producing 1 L of leachate through dilution with distilled water, while the remaining 15% constituted a residue that was then filtered. The generated filtrate was initially precipitated at pH 1, followed by filtration; then, the resultant filtrate was entirely precipitated at pH 11 using soda. This precipitate was directed to sulfuric acid till pH 1.7, washing the precipitate well with distilled water. A complete analysis was conducted on the hydrous cake, which contains La^3+^; Ce^3+^; Pr^3+^ and Nd^3+^ with 8.3, 17.9, 7.2 and 3.3% respectively. Nitric was used to thoroughly dissolve the hydrous cake of precipitate in order to prepare varied concentrations 31.2, 62.5, 125, 250, 500, 1000, 2000 mg/L, 1 and 2% for the tested experiments. The produced solutions of different concentrations were crystallized in the solution, so before the experimental work we warm it with stirring till dissolving to be used as soluble elements in nitrate solution.

### Biological activity tests

The effectiveness of rare earth elements was assessed against the white garden snail *T. pisana* under laboratory conditions. The leaf-dipping technique was used to perform this experiment. For 24 h, the snails were starving before the experiment began^28^. Four concentrations of the tested rare earth elements compound were prepared, ranging from 62.5 mg/L to 500 mg/L. The fresh leaves of lettuce were individually dipped in each concentration that has been prepared of the tested compound for one minute, while the leaves used for the control treatment was immersed solely in distilled water, and the leaves were left to dry before being presented to the tested snails. Snails were fed on the treated leaves in plastic boxes of 13 cm diameter filled with moist soil. Each box contained five healthy snails. The boxes were subsequently covered with muslin material to inhibit the snails from escaping. All treatments were repeated three times. The tested snails were examined daily for 10 days to count the dead individuals. The plastic boxes were also examined in all treatments, including the control for the first four days, to calculate the ingested lettuce leaf area and replaced them with fresh leaves. The consumption rate was estimated in each treatment by determining the areas of lettuce leaves consumed according to the method described by Mohamed and Ali^29^. Leaves treated with the concentrations used in the toxicity experiments were examined using Scanning electron microscopy (SEM). In addition, leaves treated with higher concentrations of 1000 mg/L, 1% and 2% were examined to track the crystals of rare earth elements on the surface of the treated lettuce leaves.

Regarding indicator microorganisms, by method described by (Ouis & Gamal, 2021)^30,31^ The agar diffusion method was used to apply the sample. The microorganisms were injected. The organisms were cultured overnight in nutritional agar media for bacteria and potato dextrose agar for yeast and fungus. The cultures were then placed immediately into sterile petri dishes. The concentration of the examined organisms was 106 colony-forming units per milliliter (cfu/ml). After that, a sample (100 µg/ml) was placed directly into the well of the agar plates. The inoculation plates were incubated for 24 h at the temperatures that were best for their growth. The diameter of the inhibitory zone was then measured in centimeters. The negative control (distilled water) and the positive control (standard antibiotic, Gentamicin 10 µg/disc) were used during antimicrobial testing using the agar diffusion method, which allows for accurate comparative assessment of inhibition zones. The selection of test organisms (*Staphylococcus aureus*,* Escherichia coli*,* Candida albicans*,* Aspergillus niger*, and *Bacillus cereus*) was based on their relevance as clinical and environmental indicators, providing a robust spectrum for evaluating antimicrobial efficacy. The concentration range tested was 20–2000 µg/ml, which aligns with preliminary screening thresholds in natural product antimicrobial evaluations.

### Statistical analysis

After performing the experiments, the mortality data obtained was corrected according to the Abbott equation ^32^ and subjected to probit analysis, and the values of LC_25_ and LC_50_ after ten days of treatment were determined using Finney’s probit analysis spreadsheet calculator (Version 2021)^33^. Consumption data were analyzed by using one-way ANOVA and presented as mean ± S.E. and the least significant differences at 0.05 level using Costat.exe system software (Version 6.311). The analytical methods employed for the analysis of LREEs include ICP-OES (Prism ICP - high dispersion) from Teledyne Leeman Labs, as well as a UV-vis spectrophotometer (SP-8001 UV-, Metretech Inc. version 1.02, 74 2000/10/01), which utilizes a 10 mm glass cell. The surface morphology of land snails was examined using a scanning electron microscope, specifically the JEOL-JSM-5600LV model.

## Results

### Characterization of light rare earth elements

#### Energy-dispersive X-ray spectroscopy (EDX)

**Fig. 1:**
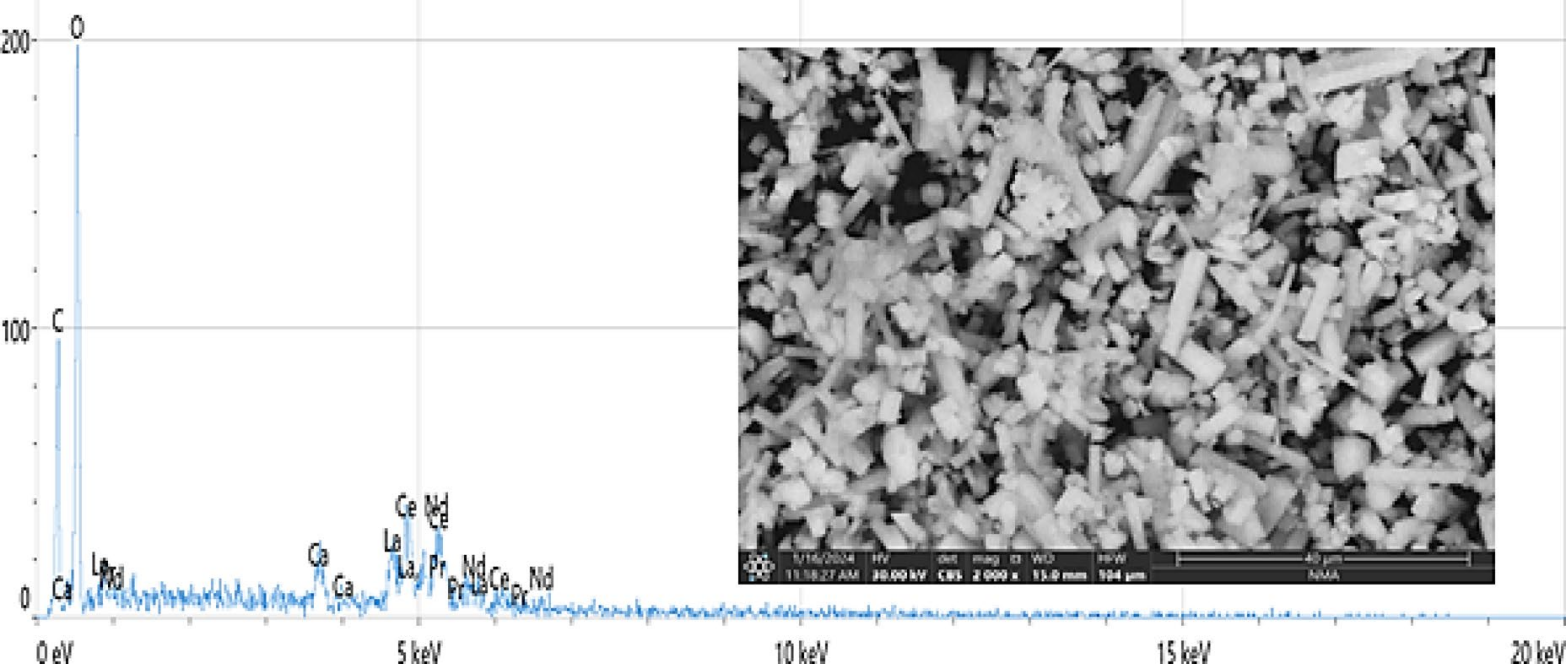
EDEX and SEM analysis of the LREEs (Light rare earth elements).

Figure 1 presents scanning electron microscope (SEM) images along with energy-dispersive X-ray (EDX) spectra of the light rare earth elements (the major material), illustrating their crystal morphology and elemental analysis. LREEs was analyzed using ICP-OES, which shows that it contains 8609, 20,213, 2575 and 16,169 of La, Ce, Pr and Nd respectively.

#### Bioactivity of different concentrations of rare earth elements against the land snail *Theba pisana*

**Fig. 2:**
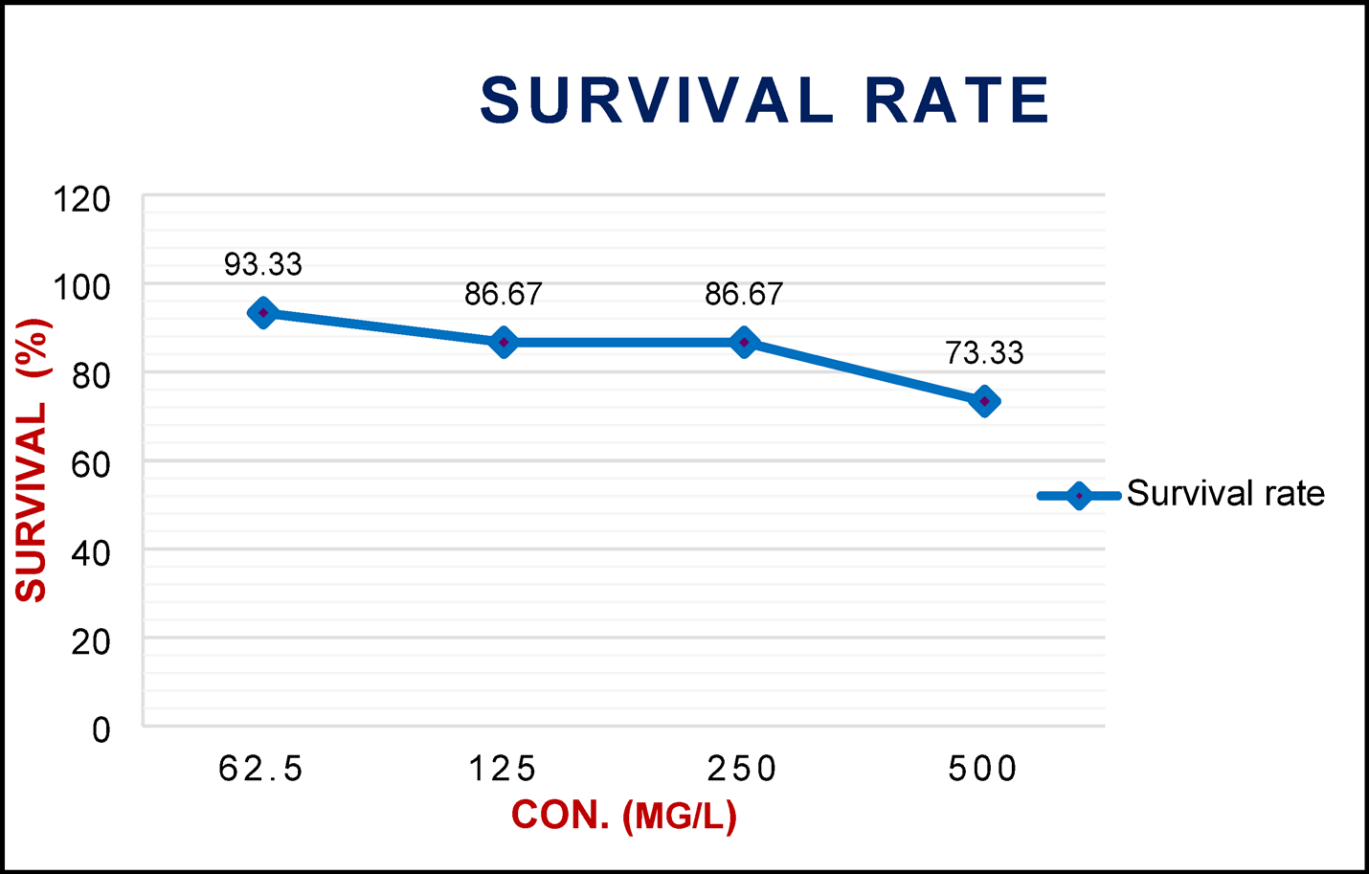
Survival rate of *Theba pisana* after exposure to different concentrations of rare earth elements under laboratory conditions.

Results illustrated in Table 1 showed the efficacy of rare earth elements based on values of lethal concentration LC_25_ and LC_50_, which were calculated for the white garden snail *Theba pisana* treated with these elements at different concentrations after ten days of treatment. The bioactivity of rare earth elements was found to increase with concentration, as evidenced by mortality rates of 6.67%, 13.33%, 13.33%, and 26.67% at concentrations of 62.5, 125, 250, and 500 (mg/L), respectively. Moreover, the calculated LC_25_ and LC_50_ after ten days of treatment were 513.70 and 3012.72 mg/L for *T. pisana* snails, respectively. In the same context, the survival rate of the exposed *T. pisana* snails to the concentrations of rare elements was significantly decreased than the control treatment (100%) (Fig. 2), with values of 73.33, 86.67, 86.67 and 93.33% at concentrations of 62.5, 125, 250 and 500 mg/L after ten days post-treatment, respectively.

**Table 1:** Efficiency and lethal concentrations of different concentrations of rare earth elements against *Theba pisana* land snails after ten days.

Conc. (mg/L)	Mortality %	LC_25_ (95% Fiducial Limits)	LC_50_ (95% Fiducial Limits)	Slope ± SE	Chi-test X^2^	Intercept
62.5	6.67	513.70 (Lower : 143.03 ) (Upper : 1844.94)	3012.72 (Lower : 838.85 ) (Upper :10820.07)	0.878 ± 0.28	0.915	1.947
125	13.33					
250	13.33					
500	26.67					
*p-value* : NS		df: 2		Fitting: Good fit		

LC_25_ = lethal concentration causing death in 25%.

LC_50_ = lethal concentration causing death in 50%.

**Fig. 3:**
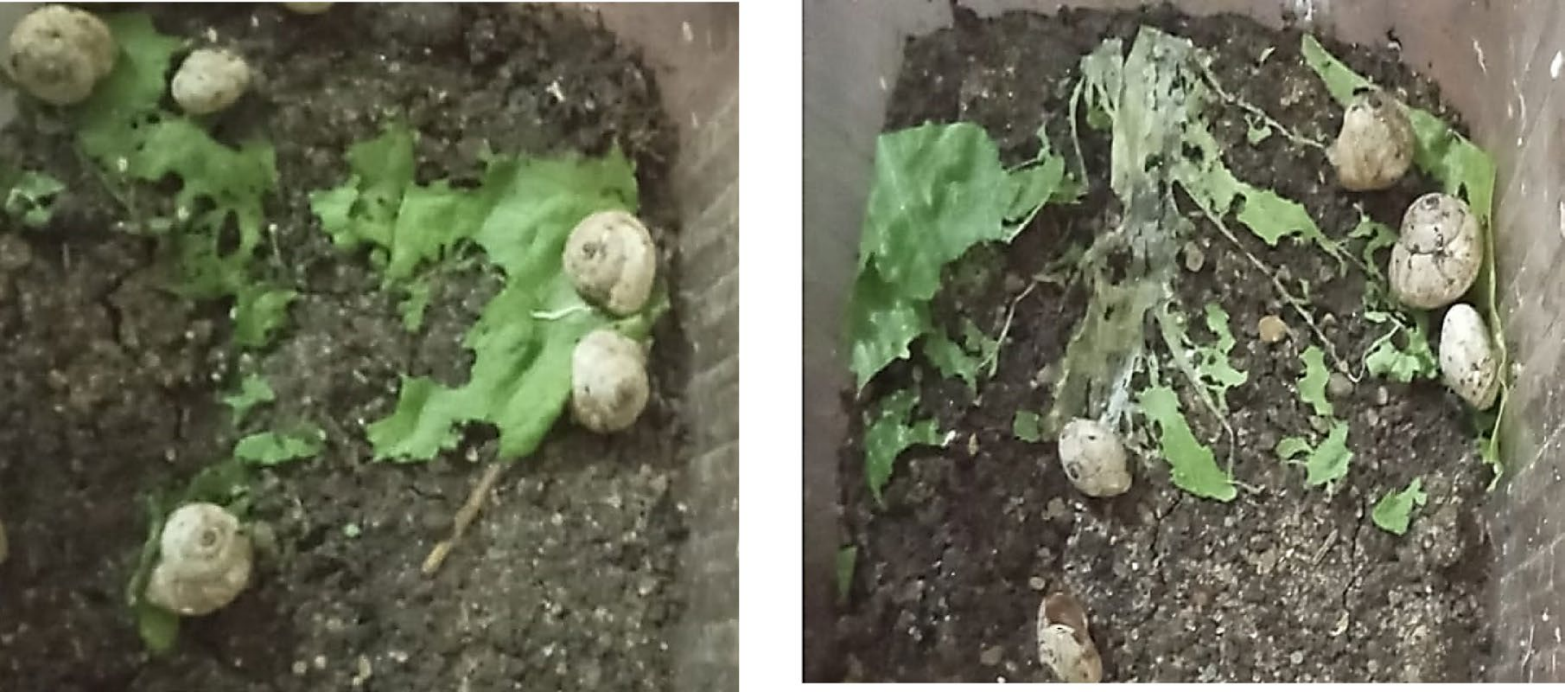
Feeding manifestations and damage of *Theba pisana* snails on treated and untreated lettuce leaves Within the replicates.

The research regarding the impacts of various concentrations of rare earth elements on the feeding ability of *T. pisana* snails was conducted by estimating the ingested leaf area and the daily consumption rate of leaves treated with these elements, compared to a control treatment under laboratory conditions, as shown in Table 2. It was observed that the ingested area and the daily consumption rate of the treated lettuce leaves decreased with increasing concentrations and longer exposure periods. The means of the ingested leaf area and the consumption rate were 60.00 ± 0.00 cm² (100.00%), 50.08 ± 2.71 cm² (83.33%), 47.33 ± 4.18 cm² (78.89%), 38.00 ± 3.61 cm² (63.33%), and 30.25 ± 6.13 cm² (50.42%) at concentrations of 0, 62.5, 125, 250, and 500 mg/L, respectively, after the first day of treatment. These values gradually decreased to 31.25 ± 0.76 cm² (52.08%), 23.00 ± 2.10 cm² (38.33%), 19.08 ± 1.76 cm² (31.81%), 12.08 ± 1.45 cm² (20.14%), and 14.00 ± 1.46 cm² (23.33%) at the same concentrations after the fourth day of treatment. Therefore, we can note that treating the leaves with low concentrations has no effect on preventing the feeding of these pests, while using these elements in high concentrations leads to their lack of appetite for snails and thus a decrease in the rate of snail consumption (Fig. 3), as it has been observed that high concentrations (1000 and 2000 mg/L) affect the freshness of treated lettuce leaves presented to the snails, and therefore the snails refrain from feeding on them.

**Table 2:** Daily food consumption of *Theba pisana* snails to the treated lettuce leaves with different concentrations of rare Earth elements under laboratory conditions.

Conc. (mg/L)	Feeding in cm^2^ per day (Mean ± SE)							
	One day		Two days		Three days		Four days	
	Ingested leaf area	Consumption (%)	Ingested leaf area	Consumption (%)	Ingested leaf area	Consumption (%)	Ingested leaf area	Consumption (%)
Control	60.00 ± 00 ^a^	100.00	45.00 ± 2.36 ^a^	75.00	32.08 ± 1.54 ^a^	53.47	31.25 ± 0.76 ^a^	52.08
62.5	50.08 ± 2.71 ^ab^	83.33	41.33 ± 2.49 ^ab^	68.89	25.58 ± 0.33 ^ab^	42.64	23.00 ± 2.10 ^b^	38.33
125	47.33 ± 4.18 ^ab^	78.89	25.75 ± 9.36 ^bc^	42.91	24.42 ± 2.82 ^ab^	40.69	19.08 ± 1.76 ^b^	31.81
250	38.00 ± 3.61 ^bc^	63.33	27.08 ± 4.72 ^bc^	45.14	17.58 ± 4.67 ^bc^	29.31	12.08 ± 1.45 ^c^	20.14
500	30.25 ± 6.13 ^c^	50.42	16.25 ± 3.75 ^c^	27.08	14.92 ± 2.48 ^c^	24.86	14.00 ± 1.46 ^c^	23.33
L.S.D. (0.05)	12.23**		16.41*		8.73**		4.95***	

* According to Duncan’s multiple range test, means that are followed by the same letter within a column do not differ significantly (*p* ≤ 0.05).

### The EDEX and SEM analysis for all the studied experiments

**Fig. 4:**
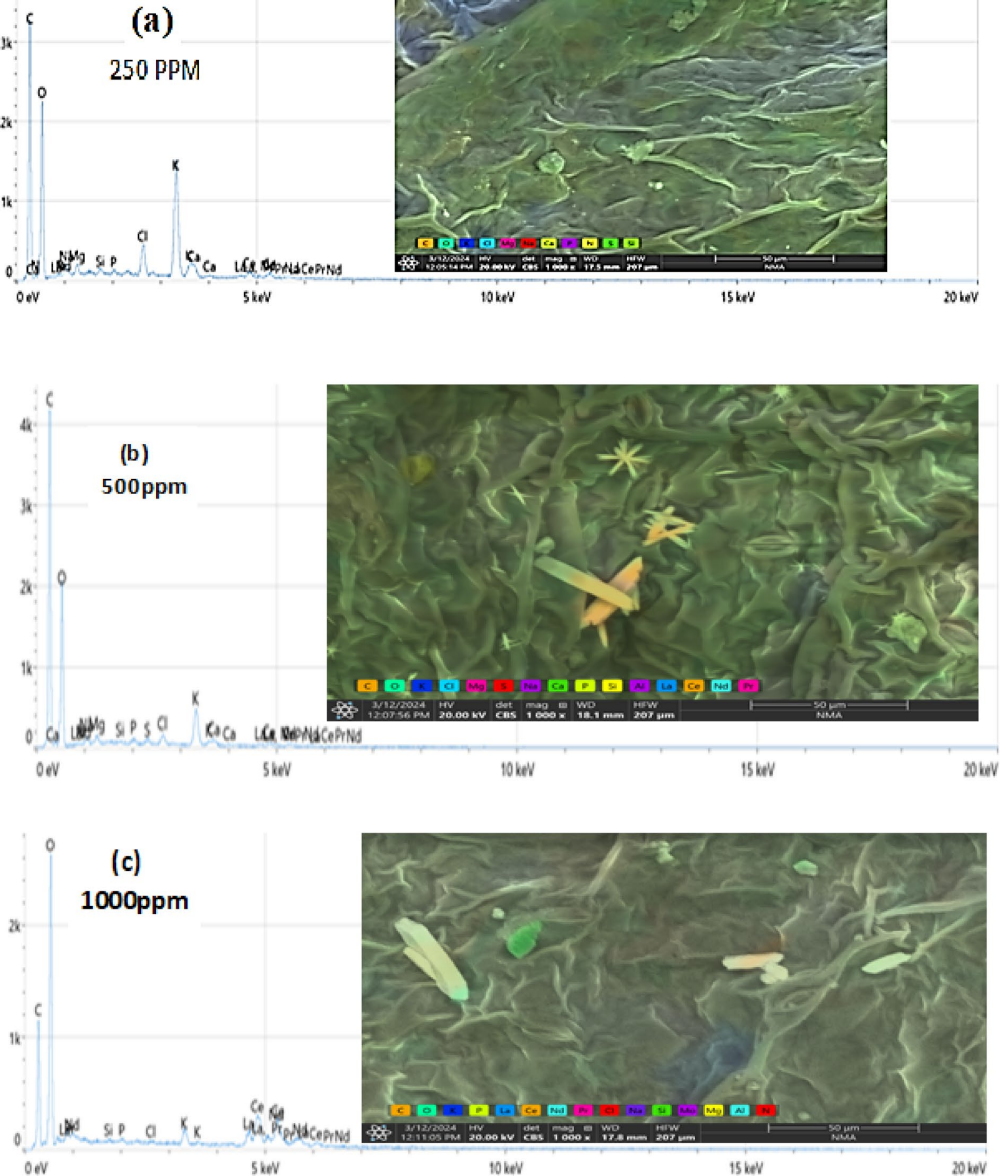

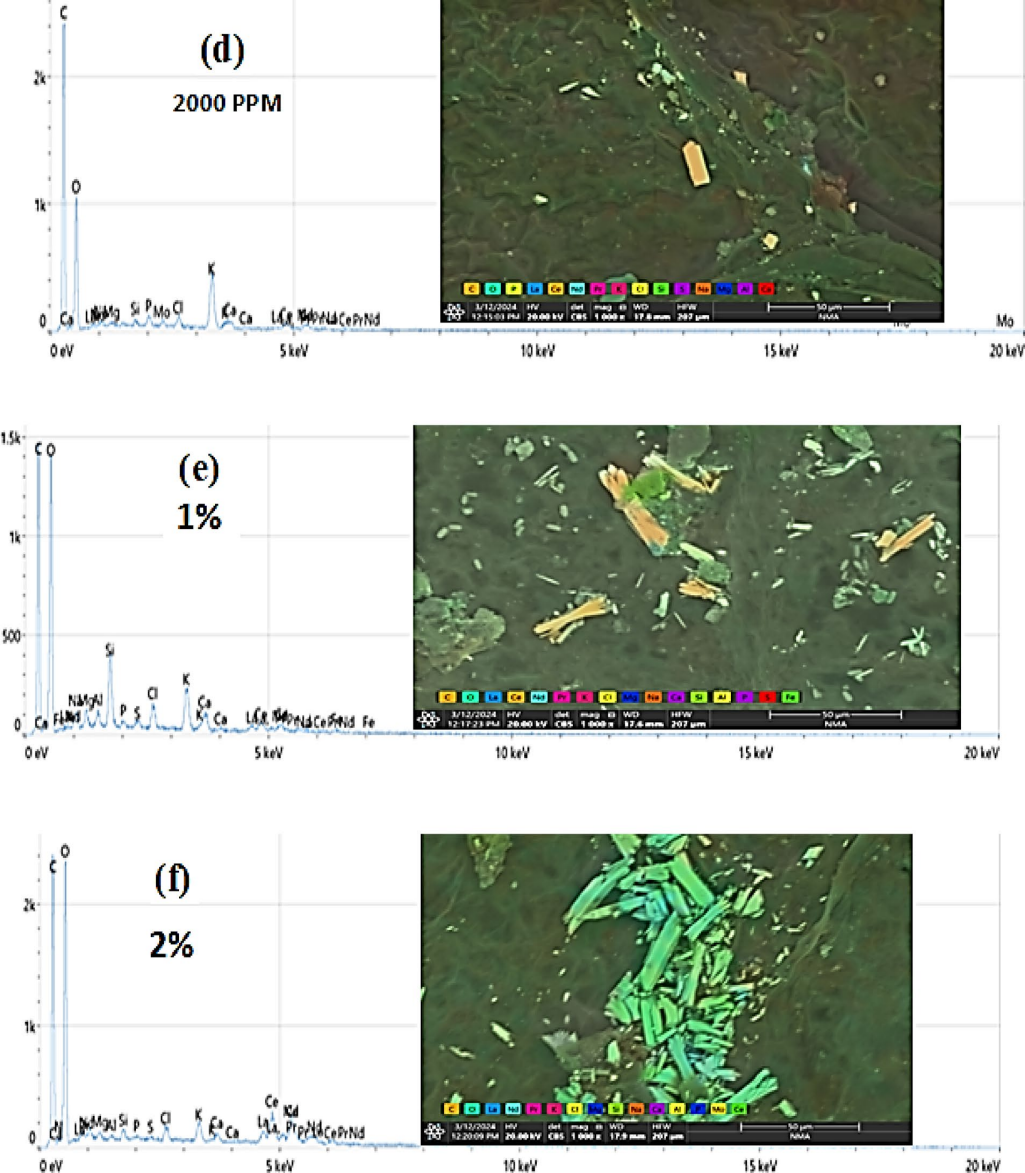
SEM images and EDEX analysis for (**a**) 250, mg/L, (**b**) 500 mg/L, (**c**) 1000 mg/L, (**d**) 2000 mg/L, (**e**) 1%, and (**f**) 2% concentration of LREEs adsorbed on lettuce leaves.

**Fig. 5:**
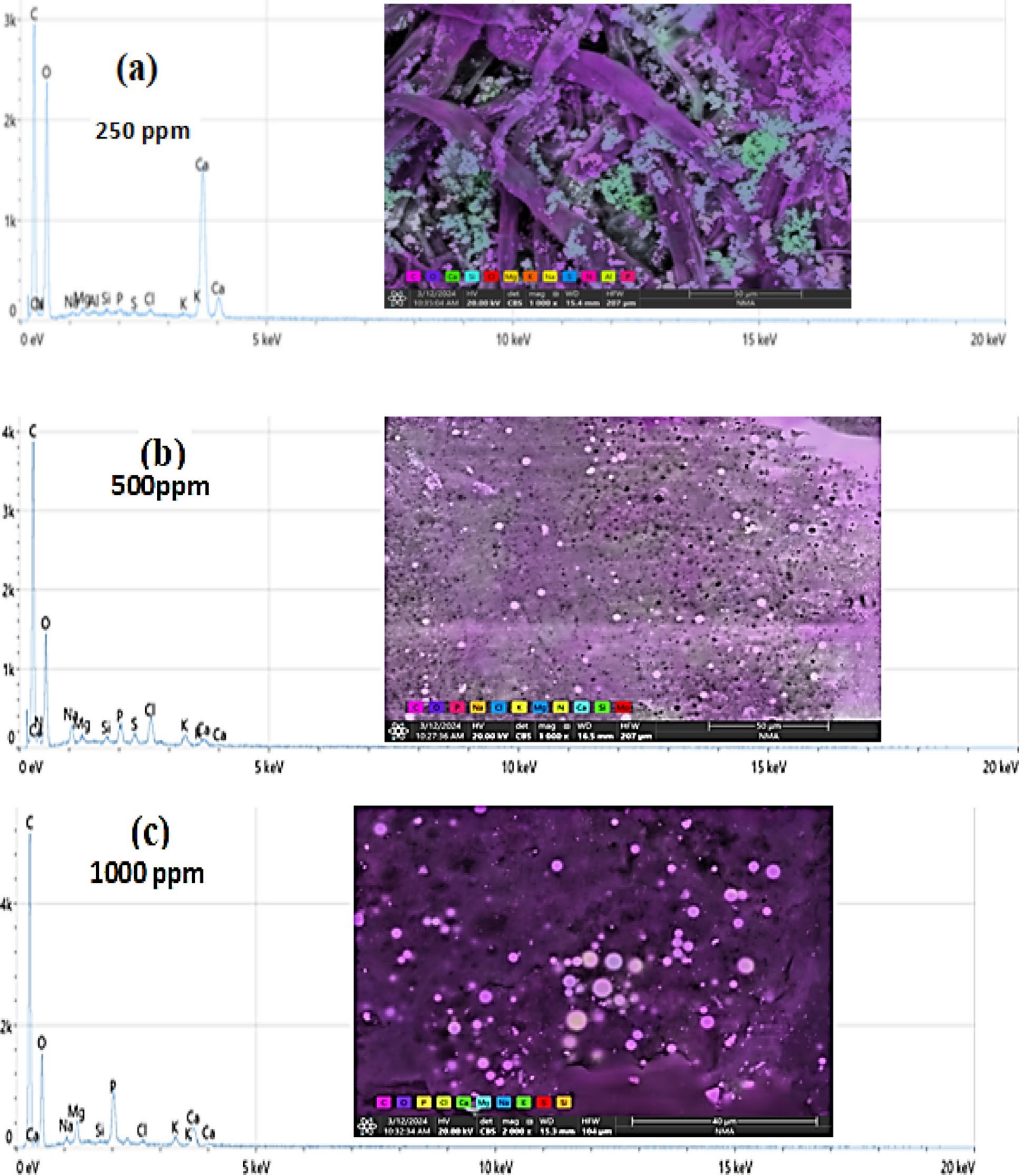

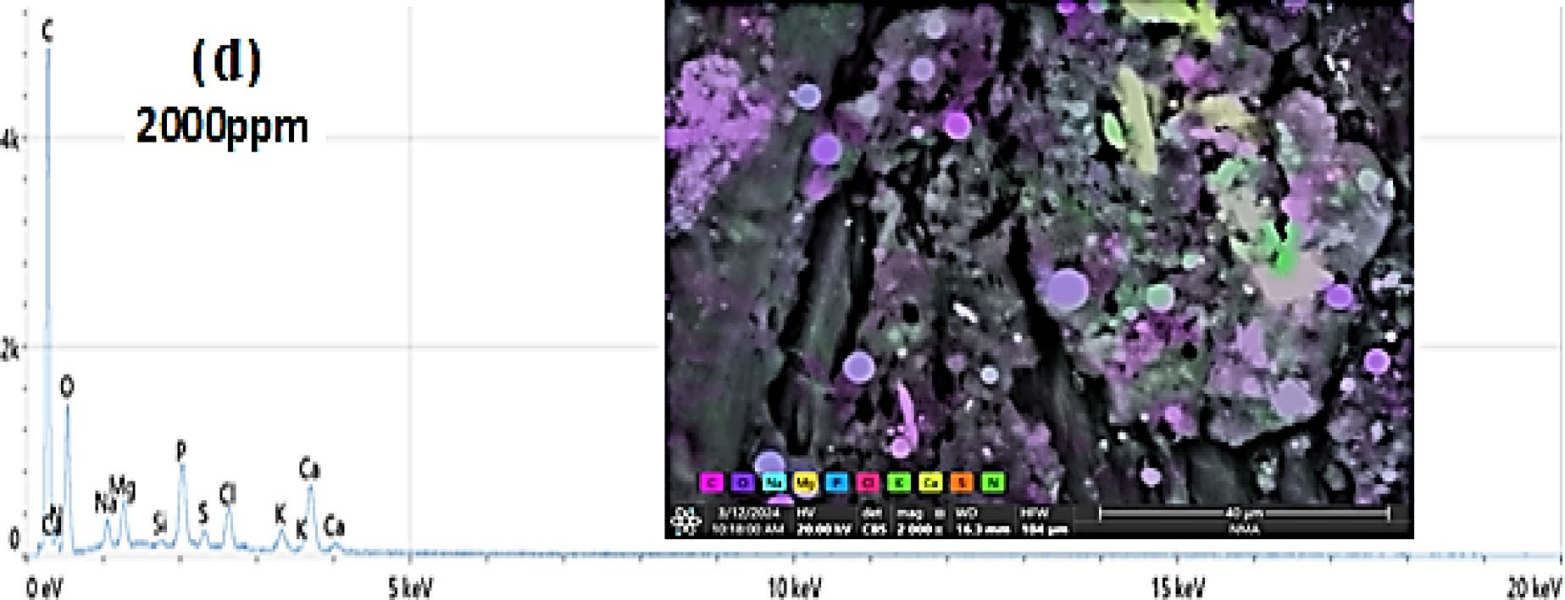
SEM images and EDEX analysis for *Theba pisana* snails after eating (**a**) 250 mg/L, (**b**) 500 mg/L, (**c**) 1000 mg/L, and (**d**) 2000 mg/L concentration of LREEs adsorbed on lettuce leaves.

The concentrations used of the 62.5 to 500 mg/L rare earth elements showed effectiveness against the tested land snails, while it was observed that when the leaves were treated with high concentrations of 1000 mg/L, 2000 mg/L, 1% and 2%, the ability of the snails to feed decreased. Therefore, the leaves treated with these concentrations were examined to trace the crystals of the tested rare earth elements. Figure 4a,b,c,d,e,f shows the lettuce leaves after immersing in different concentrations of LREEs which are 250, 500, 1000, 2000 mg/L and 1, 2% respectively. The SEM analysis show the light rare earth elements crystals adsorbed on the surface of lettuce leaves. Which show the absorption totally of low concentrations of LREEs in the leaves but in the high concentrations the leaves absorb small amounts so still the crystals of LREEs adsorbed in the surface of the leaves which appear in the SEM picture. The adsorbed lettuce leaves were subjected to the snails. *T. pisana* snails after eating the adsorbed lettuce leaves was explained in Fig. 5a,b,c,d. The *T. pisana* snails absorbed all concentrations of LREEs, so there is any presence of the LREEs in the SEM and EDEX analysis. The high concentration of LREEs made burns for the leaves because of the sensitivity of the leaves so the results was applied for the snails by concentrations of 250, 500, 1000 and 2000 mg / L not more, recommending the most effective concentrations (250 and 500 mg/L).

#### Antimicrobial activities of rare earth elements target five indicator microorganisms

**Fig. 6:**
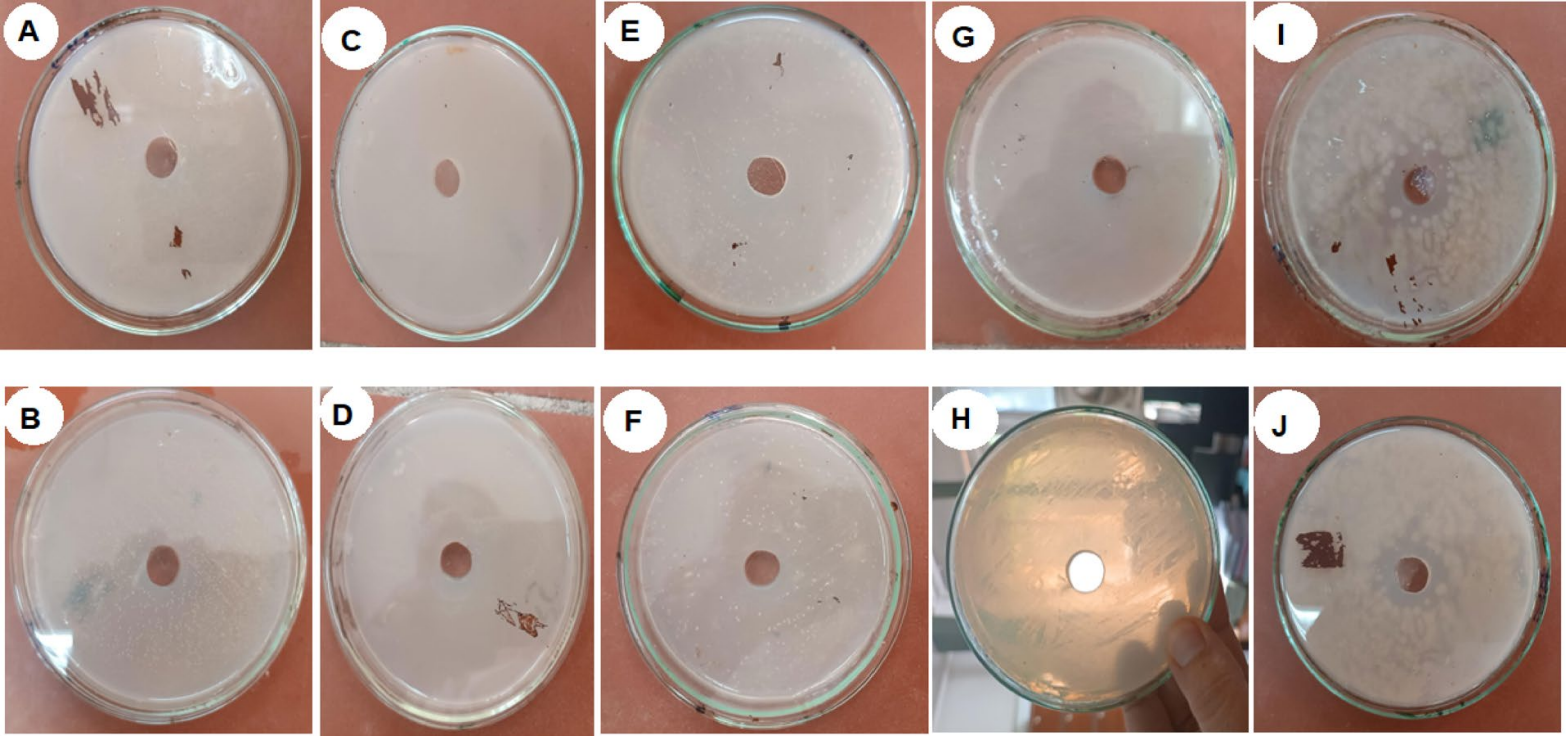
The treated strains with rare earth elements: (**A**) *Aspergillus niger* (front); (**B**) *Aspergillus niger* (back); (**C**) *Staphylococcus aureus* (front); (**D**) *Staphylococcus aureus* (back); (**E**) *Candida albicans* (front); (**F**) *Candida albicans* (back); (**G**) *Escherichia coli* (front); (**H**) *Escherichia coli* (back); (**I**) *Bacillus cereus* (front); (**J**) *Bacillus cereus* (back).

According to the methods described by Mishra & Prasad (2005) and Ouis & Gamal (2021)^30,31^ the agar diffusion method was employed to apply the samples. Overnight cultures of the tested organisms, at a concentration of 10^6^ colony-forming units per milliliter (cfu/ml), Inoculation was performed on nutrient agar for bacterial cultures and potato dextrose agar for yeast and fungal cultures, which were subsequently poured into sterile Petri dishes without delay. A sample of 100 µg/ml was subsequently introduced directly into the well of agar plates^28^. The inhibitory zone’s diameter was measured in centimeters after the inoculation plates were cultured for 24 h at their ideal growth temperatures. As illustrated in Fig. 6, the liquid form of our metal oxides demonstrates antimicrobial activity against five microorganisms that are used as indicators. Strains of indicator are *Candida albicans*,* Bacillus cereus*,* Aspergillus niger*,* Staphylococcus aureus* and *Escherichia coli*. The same procedure was carried out at another four concentrations which are 20, 40, 200, 2000 µg/ml.

**Fig. 7:**
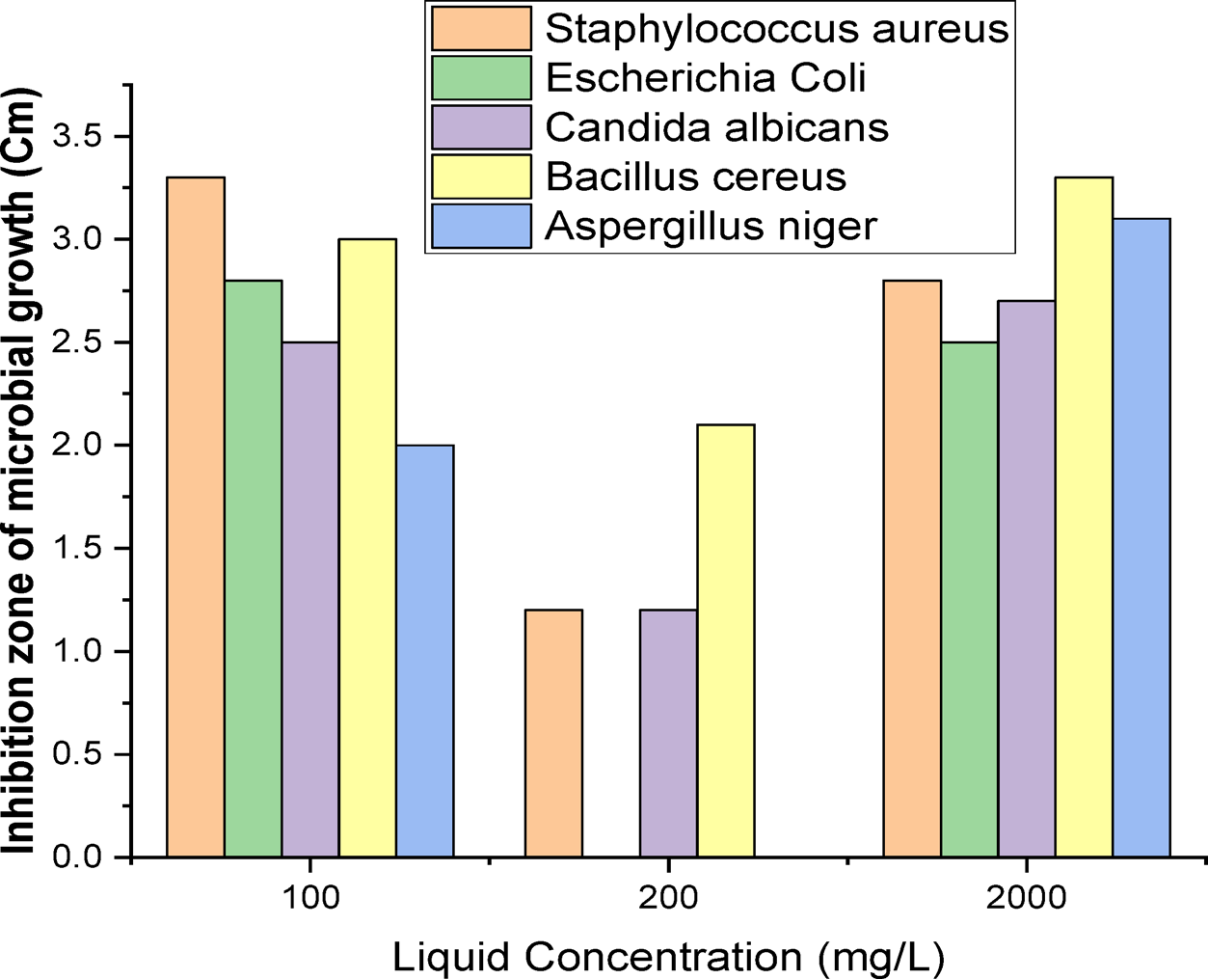
The antimicrobial activities of LREEs solution with different concentrations target by five indicator microorganisms.

From the results obtained (100 µg/ml), the inhibition zones of microbial growth measured in centimeters were as follows: *S. aureus* exhibited an inhibition zone of 3.30 cm, *E. coli* showed 2.80 cm, *C. albicans* had 2.50 cm, *B. cereus* recorded 3.00 cm, and *A. niger* demonstrated an inhibition zone of 2.00 cm. The results indicate varying degrees of antimicrobial activity against five indicator microorganisms, as evidenced by the measured inhibition zones. Specifically, *S. aureus* exhibited the largest inhibition zone at 3.30 cm, suggesting a high susceptibility to the tested antimicrobial agents. In contrast, *E. coli* and *B. cereus* displayed moderate inhibition zones of 2.80 cm and 3.00 cm, respectively, indicating a notable but lesser degree of susceptibility compared to *S. aureus*. The presence of these inhibition zones suggests that the antimicrobial agents are effective in preventing the growth of these bacteria. *C. albicans* showed an inhibition zone of 2.50 cm, which reflects a moderate response to the antimicrobial agents as well. This finding is particularly relevant as it highlights the potential for these agents to combat fungal infections as well as bacterial ones, aligning with studies that emphasize the importance of broad-spectrum antimicrobial efficacy. Lastly, *A. niger* exhibited the smallest inhibition zone at 2.00 cm, indicating a lower susceptibility compared to the other microorganisms tested. This result suggests that while some antimicrobial agents may have limited effectiveness against certain fungi. Table 3 and Fig. 7 shows the results of other concentrations 20, 40, 100, 200, 2000 µg/ml. Oxide solutions demonstrated moderate antimicrobial activity. From Table 3 higher concentration (2000 µg/ml) result in improved inhibition zones for microorganisms such as: *S. aureus* (2.8 cm) and *A. niger* (3.1 cm). So, the significant effects were found at 100 µg/ml which consider the best concentration for *S. aureus*.

**Table 3:** Antimicrobial activity of different concentrations of the oxide sample (MIC) by agar diffusion.

Sample	Inhibition zone of microbial growth (Cm)				
	Microorganisms				
	*Staphylococcus aureus*	*Escherichia coli*	*Candida albicans*	*Bacillus cereus*	*Aspergillus niger*
1 (20)	–	–	–	–	–
2 (40)	–	–	–	–	–
3 (100)	3.3	2.8	2.5	3	2
4 (200)	1.20 ± 0.13	–	1.20 ± 0.20	2.10 ± 0.14	–
5 (2000)	2.80 ± 0.18	2.50 ± 0.22	2.70 ± 0.06	3.30 ± 0.16	3.10 ± 0.06

## Discussion

Our results showed that the light rare earth elements have different degrees of activity against the tested land snails and some of the microorganisms under study. Vukašinović-Pešić et al.^34^. Research examined the bioaccumulation of twelve elements zinc, manganese, copper, aluminum, cadmium, lead, nickel, iron, chromium, lithium, selenium, and mercury in different organs of *Helix vladika* and *H. secernenda* from three sites: Biogradska Gora, Nikšić, and Malesija. The organs comprised the hepatopancreas, albumen gland, digestive tract, reproductive system, mantle, foot, and shell. The research identified notable disparities in lithium and selenium concentrations among the populations of *H. vladika* and *H. secernenda*. The concentrations of various metals (Zn, Mn, Cu, Al, Cd, Pb, Se, and Hg) exhibited significant variation among different organs, with the digestive tract and hepatopancreas demonstrating a propensity for selenium and cadmium accumulation. Sebban et al.^35^ investigated the efficacy of bioaccumulation of Pb, Cd, Zn, Cu, and Ca in *Otala* spp. snails. Their findings indicated that the snails accumulated all the analyzed components, with notable differences noted across different tissues. Principal Component and Bioaccumulation Factor studies demonstrated that *Otala* spp. serve as macroconcentrators for cadmium and microconcentrators for lead. Moreover, the identified amounts of dangerous metals (Pb and Cd) surpassed the maximum allowable limits established by European standards, with the exception of Pb at the reference station. As stated by Abdel-Halim et al.^36^ Subjecting the snail *Monacha cartusiana* to modest levels of zinc oxide nanoparticles (ZnONPs) for 14 days resulted in significant metabolic alterations attributable to oxidative stress and cellular damage. Fahmy et al.^37^ reported that ZnONPs exhibited molluscicidal action against *Biomphalaria alexandrina* snails, with an LC_50_ of 145 µg/ml. It also exhibits a harmful effect against terrestrial *Lehmannia nyctelia*^38^. According to Abdel-Azeem et al.^39^ TiO_2_NPs were toxic to the snail *Helix aspersa*, with an LC_50_ value of 544 µg/L after 24 h of exposure. Myco-synthesized SeNPs had a notable molluscicidal effect on *B. alexandrina* snails following 96 h of treatment at a dosage of 5.96 mg/L^40^. Metals such as mercury, zinc, arsenic, copper, and silver have been widely utilized in various forms as antimicrobial agents^41^. However, previous studies have not extensively explored the antimicrobial potential of rare earth elements. Given the increasing concerns regarding bacterial infections, antibiotic resistance, and the adverse effects associated with conventional antibiotics^42^ several studies have focused on the development of antibacterial materials based on metal oxides, including titanium oxide (TiO_2_)^43^ copper oxide (CuO)^44^ silver oxide (Ag_2_O)^45^ silver cations (Ag+)^46^ zero-valence silver nanoparticles (Ag0)^47^ and zinc oxide (ZnO)^48^. These materials have been applied either directly or as components within solid supports to enhance their antimicrobial efficacy. Human daily exposure to REEs can also occur due to other factors such as improper management of e-waste or high traffic volume since they are used for developing technological devices and as additives in diesel fuels. Although REE levels in the environment and food are currently present at low levels and do not possess a huge threat to human health. In some studies, they acted as antioxidants activating pathways related to numerous proteins such as SOD2, GSA, TNF-α, IL-1α, IL-8, ICAM-1, MCP-1, LC3, Beclin1, p62, caspase-9, caspase-3, BAX, Bcl-2, FAK and JPK. They can be uptaken by several cellular compartments like mitochondria, lysosomes, cytoplasm and the nucleus^49^. REE supplementation has also been shown to improve ruminal fibrolytic and proteolytic activities as well as the flavor of meat with negligible residues in edible tissue, however, the mechanism behind this action is still unclear. According to existing research, due to their poor absorption and similarity with calcium REE might exert their action locally on gut microbial populations within the gastrointestinal tract (GIT). Moreover, REE have also shown anti-inflammatory, anti-oxidative as well as immune stimulating effects. REE used as feed additives for livestock and sum up efficacy of LREEs supplementation on the performance and health of animals by comparing the findings. Till date, research with REE have shown properties that make them a promising, new and safe alternative feed additive but further exploration is recommended to optimize effects and clarify discrepancy of various results before practical proposals can be drafted^50^. according to this studies, we can say that the using of using LREEs as molluscicides and antimicrobial agents is safe and economy. Xie et al.^51^ investigated the antibacterial activity of ZnO nanoparticles against *Campylobacter jejuni* and proposed that their antibacterial effect could be attributed to cell membrane disruption and the induction of oxidative stress. Their findings demonstrated that ZnO nanoparticles led to significant morphological alterations, measurable membrane leakage, and up to a 52-fold increase in the expression of oxidative stress-related genes in *C. jejun*. The using of low concentrations of LREEs which consider more effective according to the experimental work is more economical application of using LREEs as Potential molluscicidal and antimicrobial effect. The findings support the feasibility of using LREEs as broad-spectrum antimicrobial agents, with potential applications in agricultural disease control, surface disinfection, water treatment and biocontrol formulations combining molluscicidal and antimicrobial activity. However, further research, including minimum inhibitory concentration (MIC) tests, mode-of-action assays, and field evaluations, is recommended for real-world deployment.

## Conclusions

This study provides valuable insights into the potential use of light rare earth elements (LREEs) as both molluscicides and antimicrobial agents. The results demonstrate a dose-dependent relationship between REE concentrations and mortality rates in *Theba pisana*, with lethal concentrations (LC_25_ and LC_50_) of 513.70 mg/L and 3012.72 mg/L, respectively. However, high concentrations (1000 and 2000 mg/L) also impacted the palatability of lettuce leaves, affecting the snails’ feeding behavior, with reduced consumption and leaf acceptance. SEM and EDX analyses confirm the absorption of LREEs by snails, supporting their potential use as effective molluscicides.

Moreover, the antimicrobial properties of metal oxides were confirmed through agar diffusion tests, showing broad-spectrum activity against microorganisms, especially against *Staphylococcus aureus* and *Bacillus cereus*. These findings suggest that LREEs could be developed into dual-purpose formulations, offering both molluscicidal and antimicrobial benefits. Future research should focus on optimizing these formulations for practical applications, including field trials to assess their effectiveness in natural environments and the potential for large-scale use. Additionally, further studies should explore the long-term ecological impacts of LREEs-based treatments, Taking into account the effect of high concentrations on the freshness of the leaves of some plants.

## Data Availability

The datasets generated during and/or analysed during the current study are available from the corresponding author on reasonable request.

## References

[1] Emara, E. M., Khalaf-Allah, A. S. A. & El-Sawaf, M. A. Efficacy of 2-(p-tolylamino)acetohydrazide and its Co(II), Ni(II) complexes on the shell of *Eobania vermiculata* under laboratory conditions. *Egypt. J. Agricultural Res.* **101** (4), 1019–1026. 10.21608/ejar.2023.226363.1422 (2023).

[2] Mahmoud, M., Omar, M. & Kurany, H. Ecological studies on some terrestrial snails and slugs at Sohag governorate, Egypt. *Archives Agric. Sci. J.* **4** (1), 195–204 (2021).

[3] Ibrahim, H., El-Mesalamy, A., Baghdadi, S. & Elhanbaly, R. First record of the terrestrial snail *Cochlicella acuta* (Gastropoda: pulmonata: Geomitridae) in Assiut governorate, upper Egypt. *Archives Agric. Sci. J.* **6** (1), 214–225 (2023).

[4] Sarhan, M., Abdel-Wahab, M., Aly, H. & Fouda, M. DNA barcoding of seven cone snail species from red sea Coast of Egypt. *Egypt J. Aquat. Res.* **47** (1), 93–99. 10.1016/j.ejar.2020.10.012 (2021).

[5] Abou-Elnour, B. M., El-Emam, M. A. E. W., Mahmoud, M. B., Ibrahim, W. L. & Youssef, A. A. Alternations in parasitological, biochemical and molecular parameters of *Biomphalaria alexandrina* snails, intermediate host of *Schistosoma mansoni*, induced post exposure to the proposed snail biocontrol agent *Phasmarhabditis hermaphrodita* nematode. *Asian Pac. J. Trop. Disease*. **5** (12), 957–963. 10.1016/s2222-1808(15)60964-1 (2015).

[6] Mekawey, A. A. I., Salah, A. M. & Yosri, M. A. Study on the Bio-responses of a freshwater snail (*Biomphalaria alexandrina*) to Fungal-derived compounds. *Recent. Adv. Anti-Infective Drug Discovery*. **17** (2), 139–153. 10.2174/2772434417666220610110226 (2022). 10.2174/2772434417666220610110226 35692160

[7] Ibrahim, H., El-Mesalamy, A., Baghdadi, S. & Elhanbaly, R. Species diversity and population dynamics of the prevailing land gastropod species on certain crops at Assiut governorate, Egypt. *Archives Agric. Sci. J.* **4** (1), 310–320 (2021).

[8] Mead, A. R. Helicid land mollusks introduced into North America. *Biologist* **53**, 104–111 (1971).

[9] Madejón, P. et al. The snail *Theba pisana* as an indicator of soil contamination by trace elements: potential exposure for animals and humans. *J. Sci. Food Agric.* **93**, 2259–2266 (2013). 23737085 10.1002/jsfa.6035

[10] El-Gendy, K. S., Radwan, M. A., Gad, A. F., Khamis, A. E. & Eshra, E. H. Use of multiple endpoints to investigate the ecotoxicological effects of abamectin and Thiamethoxam on *Theba pisana* snails. *Ecotoxicol. Environ. Saf.* **167**, 242–249 (2019). 30342357 10.1016/j.ecoenv.2018.10.027

[11] Radwan, M. & Gad, G. Insights into the ecotoxicological perturbations induced by the biocide abamectin in the white snail, *Theba pisana*. *J. Environ. Sci. Health Part. B *10.1080/03601234.2022.2044708 (2022). 10.1080/03601234.2022.2044708 35193456

[12] De Ley, I. T., Schurkman, J., Wilen, C. & Dillman, A. R. Mortality of the invasive white garden snail *Theba pisana* exposed to three US isolates of *Phasmarhabditis* spp (*P. hermaphrodita*, *P. californica*, and *P. papillosa*). *PLoS ONE* **15** 1–10. 10.1371/journal.pone.0228244 (2020). 10.1371/journal.pone.0228244 PMC6988931 31995592

[13] Mohamed, A. A., Said, R. M. & Zaher, E. E. Evaluation the effectiveness of *Chrysomya marginalis* maggots extract in controlling land snail *Theba pisana*. *J. Adv. Veterinary Res.* **14** (5), 819–823 (2024).

[14] Abo-Elwfa, M. M., Omar, M. M. A., El-Shamy, E. A. & Ibrahim, H. A. M. Biocontrol potential of some bacterial and fungal isolates against the terrestrial snail, *Monacha obstructa*, evaluating their laboratory and field efficiency. *Egypt. J. Biol. Pest Control* **34** (1). 10.1186/s41938-024-00788-2 (2024).

[15] Abo-Elwfa, M. M., Omar, M. M. A., El-Shamy, E. A. & Ibrahim, H. A. M. Potential molluscicidal activity of the aqueous extracts of some plants and their powders against terrestrial snail *Monacha obstructa* (L. Pfeiffer, 1842) under laboratory and field conditions. *J. Plant. Prot. Res.* 158–164. 10.24425/jppr.2024.150250 (2024).

[16] Abd El-Wahed, S. I. M. & Ibrahim, H. A. M. Molluscicidal assessment of certain toxicants: impact on biochemical alterations and electrophoretic protein patterns in *Massylaea vermiculata* (O. F. Müller, 1774) snails. *Environ. Toxicol. Pharmacol.* **113**, 104619. 10.1016/j.etap.2024.104619 (2025). 39710125 10.1016/j.etap.2024.104619

[17] Emam, N. et al. The utmost extraction of some individual rare earth elements from monazite mineral acidic leach liquor by solvent and ion exchange techniques. *Egypt. J. Chem. Vol*. **65**, 661–668. 10.21608/ejchem.2022.141255.6181 (2022). No. SI:13B.

[18] Abd El-Fatah, A. I. L. & Elashry, S. M. La (III) separation by Tri octyl phosphine oxide (Cyanex 921) based on amberlite Xad-4 chelating resin. *J. Inorg. Organomet. Polym Mater.*10.1007/s10904-022-02344-7 (2022).

[19] Rabie, K. A., Wahaab, A., Mahmoud, S. M., Hussein, K. F. & Abd El-Fatah. A. I. L. Monazite-Uranium separation and purification applying Oxalic-Nitrate- TBP extraction. *Arab. J. Nuclear Sci. Appl.* **46** (1), 30–42 (2013).

[20] Rabie, K. A., AbdElMoneam, Y. K., Abd El-Fatah, A., Demerdash, A. I. & Salem, A. R. M. Adaptation of anion exchange process to decontaminate monazite rare earth group from its uranium content. *Int. J. Res. Eng. Technol.* **03** (06), 374–382 (2014).

[21] Elwakeel, K. Z., Daher, A. M., Abd El-Fatah, A. I. L., Abd El Monem, H. & Khalil, M. M. H. Biosorption of lanthanum from aqueous solutions using magnetic alginate beads. *J. Dispersion Sci. Technology*. **38** (1), 145–151. 10.1080/01932691.2016.1146617 (2017).

[22] Abd El-Fatah, A. I. L. Purification of lanthanum oxide by magnetic nano-composite alginate beads after its concentration from acidic monazite leach liquor. *Bull. Tabbin Inst. Metall. Stud. (TIMS)*. **108** (1), 1–16. 10.21608/tims.2019.189866 (2019).

[23] Khalil, M. M. H., Atrees, M. S., Abd El Fatah, A. I. L., Salem, H. & Roshdi, R. Synthesis and application studies of chitosan acryloylthiourea derivative for the separation of rare earth elements. *J. Dispers. Sci. Technol.* **39** (4), 605–613. 10.1080/01932691.2017.1370674 (2017).

[24] El-Awady, M. E., Abdel-Ftah, A. I., L, Abdel-Wahab, S. M., Mahmoud, A. H. & Helaly, O. S. Selective precipitation of yttrium from monazite acid leach liquor by using carbonates precipitation method. *Bull. Tobbin Inst. Metallurigical Stud.* **108** (1), 30–39 (2019).

[25] Rocha, R. A., Alexandrov, K. & Scott, C. Rare Earth elements in biology: from biochemical curiosity to solutions for extractive industries. *Microb. Biotechnol.* **17** (6). 10.1111/1751-7915.14503 (2024). 10.1111/1751-7915.14503 PMC11146143 38829373

[26] Ibrahim, H. A. M., El-Mesalamy, A. F., Baghdadi, S. A. & Elhanbaly, R. Histopathological effects of methomyl and crude extracts of *Jatropha curcas* against the terrestrial snail, *Monacha obstructa* (Gastropoda:Hygromiidae). *Chem. Biol. Technol. Agric.* **9**, 65. 10.1186/s40538-022-00330-2 (2022).

[27] Abd El-Fatah, A. I. L. An innovative method for Egyptian monazite mineral digestion by sulfuric acid. *Int. J. Sci. Res. (IJSR)*. **10** (3), 1194–1202. 10.21275/sr21316221646 (2021).

[28] Shetaia, Z. S. Integrated control of land snails pests in the fields of Sharkia governorate. Ph.D. Thesis in agricultural of zoology and nematology. *Al-Azhr University* 147 (2005).

[29] Mohamed, M. I. & Ali, R. F. Effect of photoperiods· on· the rate of food consumption by the land snail *Monacha cartusiana* (Muller). *J. Plant. Prot. Pathol.* **30** (11), 7147–7152 (2005).

[30] Mishra, V. & Prasad, D. N. Application of in vitro methods for selection of *Lactobacillus casei* strains as potential probiotics. *Int. J. Food Microb.* **103** (1), 109–115. 10.1016/j.ijfoodmicro.2004.10.047 (2005). 10.1016/j.ijfoodmicro.2004.10.047 16040148

[31] Ouis, M. & Gamal, A. A. Role of silver ions in Na₂O-CaF₂-P₂O₅ host glass and its corresponding glass-Ceramic: searching for antibacterial Behaviour- supplemented by spectral, optical, FTIR and SEM investigations. *Egypt. J. Chem.* **64**, 5345–5355 (2021).

[32] Abbott, W. S. A method of computing the effectiveness of an insecticide. *J. Econ. Entomol.* **18**, 265–267 (1925). 3333059

[33] Mekapogu, A. R. Finney’s probit analysis spreadsheet calculator (Version 2021). https://probitanalysis.wordpress.com/ (2021).

[34] Vukašinović-Pešić, V. et al. Toxic elements and mineral content of different tissues of endemic edible snails (*Helix vladika* and *H. secernenda*) of Montenegro. *Foods* **9** (6), 731. 10.3390/foods9060731 (2020). 32503124 10.3390/foods9060731 PMC7353529

[35] Sebban, H. et al. Trace element bioaccumulation in the edible milk snail (*Otala lactea*) and Cabrilla (*Otala punctata*) in marrakech, Morocco. *Appl. Ecol. Environ. Res.* **20** (1), 875–892. 10.15666/aeer/2001_875892 (2022).

[36] Abdel-Halim, K. Y., Osman, S. R. & Abdou, G. Y. In vivo evaluation of oxidative stress and biochemical alteration as biomarkers in glass clover snail, *Monacha cartusiana* exposed to zinc oxide nanoparticles. *Environ. Pollut.* **257**, 113120. 10.1016/j.envpol.2019.113120 (2020). 31753629 10.1016/j.envpol.2019.113120

[37] Fahmy, S. R., Abdel-Ghaffar, F., Bakry, F. A. & Sayed, D. A. Ecotoxicological effect of sublethal exposure to zinc oxide nanoparticles on freshwater snail *Biomphalaria alexandrina*. *Arch. Environ. Contam. Toxicol.* **67** (2), 192–202. 10.1007/s00244-014-0020-z (2014). 24736985 10.1007/s00244-014-0020-z

[38] Mohammad, W. A., Ali, S. M., Farhan, N. & Said, S. M. The toxic effect of zinc oxide nanoparticles on the terrestrial slug *Lehmannia nyctelia* (Gastropoda-Limacidae). *J. Basic Appl. Zool.* **82** (1). 10.1186/s41936-021-00214-1 (2021).

[39] Abdel-Azeem, H. H., Osman, G. Y. & Mohamed, A. H. Potential toxic effects of titanium oxide (TiO_2_) nanoparticles on the biological, biochemical, and histological aspects of the land snail *Helix aspersa*. *Environ. Sci. Pollut. Res.* **30** (32), 78127–78138. 10.1007/s11356-023-27666-y (2023). 10.1007/s11356-023-27666-y PMC10313540 37266786

[40] Morad, M. Y. et al. Myco-Synthesized molluscicidal and larvicidal selenium nanoparticles: A new strategy to control *Biomphalaria alexandrina* snails and larvae of *Schistosoma mansoni* with an in Silico study on induced oxidative stress. *J. Fungi*. **8** (3), 262. 10.3390/jof8030262 (2022). 10.3390/jof8030262 PMC8952376 35330264

[41] Mittapally, S., Taranum, R. & Parveen, S. Metal ions as antibacterial agents. *J. Drug Delivery Therapeutics*. **8** (6-s), 411–419. 10.22270/jddt.v8i6-s.2063 (2018).

[42] Roy, A., Butola, B. S. & Joshi, M. Synthesis, characterization and antibacterial properties of novel nano-silver loaded acid activated montmorillonite. *Appl. Clay Sci.* **146**, 278–285. 10.1016/j.clay.2017.05.043 (2017).

[43] Yang, D., Yuan, P., Zhu, J. X. & He, H. P. Synthesis and characterization of antibacterial compounds using montmorillonite and chlorhexidine acetate. *J. Therm. Anal. Calorim.* **89** (3), 847–852. 10.1007/s10973-006-8318-3 (2007).

[44] Özdemir, G. & Yapar, S. Preparation and characterization of copper and zinc adsorbed cetylpyridinium and N-lauroylsarcosinate intercalated montmorillonites and their antibacterial activity. *Colloids Surf., B*. **188**, 110791. 10.1016/j.colsurfb.2020.110791 (2022). 10.1016/j.colsurfb.2020.110791 31955019

[45] Khezerlou, A., Alizadeh-Sani, M., Azizi-Lalabadi, M. & Ehsani, A. Nanoparticles and their antimicrobial properties against pathogens including bacteria, fungi, parasites and viruses. *Microb. Pathog.* **123**, 505–526. 10.1016/j.micpath.2018.08.008 (2018). 30092260 10.1016/j.micpath.2018.08.008

[46] Roy, A., Joshi, M., Butola, B. S. & Malhotra, S. Antimicrobial and toxicological behavior of montmorillonite immobilized metal nanoparticles. *Mater. Sci. Engineering: C*. **93**, 704–715. 10.1016/j.msec.2018.08.029 (2018). 10.1016/j.msec.2018.08.029 30274104

[47] Cruces, E. et al. Copper/Silver bimetallic nanoparticles supported on aluminosilicate geomaterials as antibacterial agents. *ACS Appl. Nano Mater.* **5** (1), 1472–1483. 10.1021/acsanm.1c04031 (2022).

[48] Ghadiri, M., Chrzanowski, W. & Rohanizadeh, R. Biomedical applications of cationic clay minerals. *RSC Adv.* **5** (37), 29467–29481. 10.1039/c4ra16945j (2015).

[49] Brouziotis, A. A. et al. Toxicity of rare earth elements: an overview on human health impact. *Front. Environ. Sci.* **10**10.3389/fenvs.2022.948041 (2022).

[50] Tariq, H. et al. Perspectives for rare Earth elements as feed additive in livestock — A review. *Asian-Australasian J. Anim. Sci.* **33** (3), 373–381. 10.5713/ajas.19.0242 (2019). 10.5713/ajas.19.0242 PMC7054624 31480174

[51] Xie, Y., He, Y., Irwin, P. L., Jin, T. & Shi, X. Antibacterial activity and mechanism of action of zinc oxide nanoparticles against *Campylobacter jejuni*. *Appl. Environ. Microbiol.* **77** (7), 2325–2331. 10.1128/aem.02149-10 (2011). 21296935 10.1128/AEM.02149-10 PMC3067441

